# SIMPL Enhancement of Tumor Necrosis Factor-α Dependent p65-MED1 Complex Formation Is Required for Mammalian Hematopoietic Stem and Progenitor Cell Function

**DOI:** 10.1371/journal.pone.0061123

**Published:** 2013-04-22

**Authors:** Weina Zhao, Erin Breese, Allison Bowers, Jonathan Hoggatt, Louis M. Pelus, Hal E. Broxmeyer, Mark Goebl, Maureen A. Harrington

**Affiliations:** 1 Department of Biochemistry and Molecular Biology, Indiana University School of Medicine, Indianapolis, Indiana, United States of America; 2 Department of Microbiology and Immunology, Indiana University School of Medicine, Indianapolis, Indiana, United States of America; New York University, United States of America

## Abstract

Significant insight into the signaling pathways leading to activation of the Rel transcription factor family, collectively termed NF-κB, has been gained. Less well understood is how subsets of NF-κB-dependent genes are regulated in a signal specific manner. The SIMPL protein (signaling molecule that interacts with mouse pelle-like kinase) is required for full Tumor Necrosis Factor-α (TNFα) induced NF-κB activity. We show that SIMPL is required for steady-state hematopoiesis and the expression of a subset of TNFα induced genes whose products regulate hematopoietic cell activity. To gain insight into the mechanism through which SIMPL modulates gene expression we focused on the *Tnf* gene, an immune response regulator required for steady-state hematopoiesis. In response to TNFα SIMPL localizes to the *Tnf* gene promoter where it modulates the initiation of *Tnf* gene transcription. SIMPL binding partners identified by mass spectrometry include proteins involved in transcription and the interaction between SIMPL and MED1 was characterized in more detail. In response to TNFα, SIMPL is found in p65-MED1 complexes where SIMPL enhances p65/MED1/SIMPL complex formation. Together our results indicate that SIMPL functions as a TNFα-dependent p65 co-activator by facilitating the recruitment of MED1 to p65 containing transcriptional complexes to control the expression of a subset of TNFα-induced genes.

## Introduction

NF-κB controlled genes are a defining feature of the immune system, regulating both immune cell development and function. Proto-typical NF-κB is a homo- or hetero-dimer of members of the Rel family of DNA binding proteins that in mammals includes RelA (p65), c-rel, RelB, p105 (the precursor of p50), and p100 (the precursor of p52). Analysis of endogenous gene expression in mouse embryo fibroblasts (MEFs) derived from mice lacking individual Rel family members has revealed that DNA binding site specificity of the c-rel/NF-κB family members is not merely regulated by the binding site sequence [1]. Instead, a combinatorial model for NF-κB controlled gene expression has been proposed in which additional stimulus-specific factors are required.

Several distinct signaling pathways have been identified that control NF-κB activity [2], [3] The canonical pathway, through which inflammatory cytokines and bacterial products activate NF-κB, involves activation of a preformed complex containing IκB kinase-α (IKKα), IκB kinase-β (IKKβ) and IKKγ/NEMO, a non-enzymatic scaffold protein. Activated IKKα and IKKβ phosphorylate two key serine residues located amino terminal to the ankyrin repeat region in IκBα and IκBβ [4]–[8]. Phospho-IκB is ubiquitylated on lysine residues near the phosphorylated residues, leading to removal of IκB from the NF-κB (p65/p50) containing complex via proteolysis. IκB degradation allows NF-κB nuclear relocalization leading to changes in gene transcription. Analysis of mouse embryo fibroblasts derived from IKKα^−/−^ and IKKβ^−/−^ mice suggest that IKKα controls basal NF-κB activity whereas IKKβ controls cytokine induced NF-κB activity [9]–[14].

As a model for stimulus-induced NF- κB dependent gene expression we have utilized tumor necrosis factor-α (TNFα) to gain mechanistic insight into how signal specific changes NF-κB dependent gene transcription are achieved. TNFα is a homeostatic regulator of the inflammatory response, capable of activating as well as limiting its extent and duration [15]. Studies in mice lacking individual Rel family members have linked the p50/p65 heterodimer to the control of genes whose products regulate the inflammatory response, an essential immune system function (reviewed in [16]). The pathophysiology of diseases such as diabetes, atherosclerosis and some cancers is associated with chronic activation of the inflammatory response that in turn exacerbates the disease process [17]–[19]. Insight into mechanisms that enable the selective control of subsets of NF-κB dependent genes should identify targets for the development of innovative therapeutics to treat chronic inflammatory diseases.

In previous studies we identified a novel regulatory protein termed SIMPL (substrate that interacts with mouse pelle-like kinase; also known as interleukin-1 receptor associated kinase 1 binding protein 1, IRAK1BP1). SIMPL specifically regulates TNFα-, but not interleukin-1 (IL-1)-, induced NF-κB activity [20], [21]. Independent of TNFα-induced NF-κB nuclear localization, TNFα signaling through the type I TNFα receptor (TNF RI) leads to the phosphorylation and nuclear accumulation of SIMPL, [20], [22]. TNFα stimulates the formation of endogenous, nuclear SIMPL-p65 complexes, where SIMPL synergistically enhances p65 trans-activation activity thereby functioning as a p65 co-activator [20]. SIMPL has no activity in the absence of p65 and has no effect upon the activity of transcription factors, such as c-Jun or C/EBPβ, that frequently have binding sites in TNFα-dependent genes [20]. These properties of SIMPL enable us to address a fundamental question in the NF-κB field. A number of agents activate NF-κB leading to overlapping but not identical changes in the expression of ∼200 known NF-κB genes. To date the mechanism through which selective differences in expression can be mediated is unclear. Our results suggest that one mechanism by which this is achieved is through signal specific co-activators of p65. Herein, TNFα-induced SIMPL-dependent genes are identified and the mechanism by which SIMPL exerts its co-activator function is characterized. Our studies reveal that SIMPL functions as a p65 co-activator by facilitating the recruitment of MED1 to p65 containing transcriptional complexes to control the expression of genes involved in hematopoiesis and hematopoietic stem and progenitor cell function.

## Materials and Methods

### Plasmids, antibodies and growth factors

Flag-tagged SIMPL, amino acids 1-259 of the mouse SIMPL protein, was expressed in the pFLAG-CMV-2™ expression vector (Sigma Aldrich, St. Louis, MO). SIMPL antibody, generated by immunizing rabbits against full length recombinant SIMPL protein, was affinity purified against the recombinant protein to enrich for SIMPL specific antibody (Cocalico, Denver, PA). The p65 antibody (06-418) was from Millipore (Billerica, MA); MED1 antibody (ab60950) was from Abcam (Cambridge, MA); antibody to RNAPIIa (8WG16) and to RNAPIIo (H14) were from Covance (Princeton, NJ). Anti-FLAG® M2 monoclonal antibody (F3165) and β-actin antibody (AC-15) (A5441) were obtained from Sigma Aldrich (St. Louis, MO). Goat anti-mouse IgG F(ab′)_2_ fragment specific-HRP (115-36-006) was obtained from Jackson ImmunoResearch Laboratories, Inc. (West Grove, PA). Recombinant human (rh) TNFα and recombinant mouse SCF were from PeproTech Inc. (PeproTech, Rocky Hill, NJ); recombinant human erythropoietin (Amgen); pokeweed mitogen spleen cell conditioned media was from StemCell Technologies (Vancouver, British Columbia, Canada).

### Cell culture and transfection

Human embryonic kidney epithelial cells (HEK 293; ATCC® number CRL-1573™) were propagated in Dulbecco's Modified Eagle's Medium (DMEM; Cellgro®, Mediatech, Inc., Herndon, VA) containing 10% heat-inactivated fetal bovine serum (FBS; Hyclone, Logan, UT) supplemented with L-glutamine and penicillin/streptomycin. Mouse embryo fibroblasts (MEFs) were propagated in Basal Medium Eagle (BME; Sigma-Aldrich, St. Louis, MO) containing 10% heat-inactivated FBS supplemented with L-glutamine and penicillin/streptomycin. All cells were maintained at 37°C with 5% carbon dioxide. Plasmid constructs were introduced into cells as CaPO_4_ precipitates.

### Immunocomplexing assays

Cell monolayers were washed three times with ice cold phosphate buffered saline (PBS). Immunoprecipitation (IP) lysis buffer (10 mM HEPES, pH 7.4, 150 mM NaCl, 5 mM EDTA, 1% Triton X-100) containing protease inhibitors (Complete Protease Inhibitor Cocktail, Roche, Indianapolis, IN) was added directly to the cell monolayer. Cell monolayers were incubated with lysis buffer (4°C, 20 min), lysates were collected and centrifuged (20 min, 16,000× g, 4°C) to remove cellular debris. Nuclear extracts were generated as described by Schreiber et al., [23]. Briefly, PBS rinsed cell monolayers collected by centrifugation (30 sec., 16,000× g, RT), were resuspended and incubated for 15 min (4°C) in a hypotonic lysis buffer (10 mM Hepes, pH 7.9; 10 mM KCl, 0.1 mM EDTA, 0.1 mM EGTA, 1 mM DTT and 0.5 mM PMSF) containing protease inhibitors. Following the addition of NP-40 cell suspensions were centrifuged (30 sec., 16,000× g, RT), and nuclear pellets were resuspended in nuclear extract buffer (20 mM Hepes, pH 7.9, 0.4 M NaCl, 1 mM EDTA, 1 mM EGTA, 1 mM DTT, 1 mM PMSF) containing protease inhibitors. Following a 15 min incubation (4°C) with repeated vortexing, insoluble materials were removed by centrifugation (20 min, 16,000× g, 4°C). Supernates were adjusted with hypotonic lysis buffer to a final salt concentration of 125 mM.

Supernates (whole cell or nuclear) were incubated (1 hour, 4°C) with protein-A sepharose (Sigma-Aldrich, St. Louis, MO), centrifuged to pellet the sepharose beads and supernates were incubated with protein-A sepharose beads and the indicated antibody (amount according to the manufacturer's specifications; 16 h, 4°C) overnight. Materials bound to the protein-A sepharose beads were collected by centrifugation (325× g, 2 min, 4°C), supernates were removed and pelleted materials were subject to four cycles of resuspension (10 mM HEPES, pH 7.4, 150 mM NaCl, 5 mM EDTA, 0.1% Triton X-100)/centrifugation. Following removal of the final supernate Laemmli buffer (Bio-Rad, Hercules, CA) was added to the pelleted materials. Denatured samples were subjected to Western analysis as described previously [20]. All immunocomplexing assays were repeated at least three times.

### Generation and analysis of TAP-tagged SIMPL

The wild-type SIMPL coding sequence was subcloned into the pNTAP-A vector (InterPlay™ Mammalian TAP system (Stratagene, La Jolla, CA). The TAP-tagged wild-type SIMPL construct was sequenced, expressed in HEK 293 cells to confirm production of the correctly sized fusion protein and to confirm that the SIMPL fusion protein was capable of inducing the activity of an NF-κB dependent reporter when overexpressed (data not shown). To generate TAP-SIMPL containing protein complexes, HEK 293 cells were transfected with empty vector or the TAP-tagged SIMPL construct, twenty-four hours later cell monolayers were rinsed and harvested in PBS. Whole cell lysates were generated using the InterPlay™ lysis buffer followed by three successive freeze/thaw cycles. Lysates were incubated with a streptavidin resin to enrich for TAP-SIMPL containing complexes. The streptavidin resin was washed, and bound complexes were eluted from the resin by the addition of biotin. Eluted complexes were subsequently incubated with a calmodulin resin to further enrich for TAP-SIMPL containing complexes. The calmodulin resin was washed, and bound complexes were eluted from the resin by calcium chelation. Isolated protein complexes were subsequently subjected to TCA/acetone precipitation. To identify TAP-SIMPL containing complex components, the isolated protein complexes were submitted to the Indiana Centers for Applied Protein Sciences (INCAPS) for analysis by mass spectrometry. Briefly, protein samples were reduced, alkylated, and digested with trypsin. Tryptic peptides were analyzed by LTQ ion-trap LC-MS/MS, and data were collected. Fragmentation data were analyzed using SEQUEST™ and experimental MS/MS were searched against an in silico tryptic digest of the human IPI (version 13) non-redundant protein database. Peptide probabilities (PeptideProphet; [24]) were assigned to peptides identified by SEQUEST analysis. Peptides were grouped into proteins using ProteinProphet [25]. Proteins with a probability of ≥0.6 were considered for further analysis.

### Chromatin immunoprecipitation (ChIP) assays

The indicated MEFs were plated in 150 mm tissue culture dishes and grown to 80–90% confluency. Following a seventy-two hour transfection with the indicated constructs and/or treatment with rhTNFα (10 ng/mL) for 45 min, cell monolayers were gently washed two times with PBS containing 1 mM MgCl_2_. Cell monolayers were subjected to cross-linking with 1% formaldehyde at room temperature (RT; 20 min) and the cross-linking was terminated by the addition of glycine (0.125 M final concentration). Cell monolayers were then rinsed twice with ice-cold PBS/MgCl_2_. Cells were harvested in 30 ml PBS and collected by centrifugation (735× g, 1 min, RT). Cell pellets were resuspended in 3 ml cell lysis buffer (85 mM KCl, 0.5% NP40, 5 mM HEPES (KOH), pH 8.0 and protease inhibitors) and incubated on ice for 10 minutes. Nuclei were collected by centrifugation (1,310× g, 5 min, 4°C), and supernates were discarded. Nuclei were resuspended in 3 ml nuclear lysis buffer (1% SDS, 10 mM EDTA, 50 mM Tris, pH 8.0) and incubated on ice for 10 minutes. To shear DNA the nuclei were sonicated using 20×10 second pulses followed by a 30 sec break on ice. Sonicated samples were centrifuged (13,800× g, 10 min, 4°C) to remove insoluble materials. For individual ChIP assays, an appropriate volume of lysate was mixed with ChIP Dilution Buffer (165 mM NaCl, 0.01% SDS, 1.1% Triton X-100, 1.2 mM EDTA, 16.7 mM Tris-HCl, pH 8.0 and protease inhibitors). A small amount of the diluted lysate was from for each sample was reserved and served as the input control. Diluted lysates were incubated with salmon sperm DNA saturated protein A agarose beads (Upstate, Charlottesville, VA) for one hour (4°C) to reduce non-specific binding. Agarose beads were collected by centrifugation (1,310× g, 1 min, 4°C), and supernates were transferred to a clean Eppendorf tube. Samples were incubated overnight (4°C) with 4 µg of the indicated antibody; one sample per condition did not receive antibody to serve as a negative control. To facilitate isolation of antibody containing complexes, salmon sperm DNA saturated protein A agarose beads was added to each sample and samples were incubated for an additional four hours (4°C). Agarose beads were collected by centrifugation (1,310× g, 1 min, 4°C), and supernates were removed. Pelleted materials were subjected to two cycles of resuspension/centrifugation sequentially in: a low-salt buffer (150 mM NaCl, 0.1% SDS, 1% NP-40, 1 mM EDTA, 50 mM Tris-HCl), a high-salt buffer (500 mM NaCl, 0.1% SDS, 1% NP-40, 1 mM EDTA, 50 mM Tris-HCl), a LiCl buffer (250 mM LiCl, 0.1% SDS, 1% NP-40, 1 mM EDTA, 1% Deoxycholic Acid, 50 mM Tris-HCl), and finally in TE buffer (10 mM Tris-HCl, 0.25 mM EDTA). The final agarose bead containing pellet was resuspended in Elution Buffer (1% SDS, 0.1 M NaHCO_3_) and incubated for 15 minutes at RT. Agarose beads were collected by a brief centrifugation and eluates were transferred to a clean eppendorf tube. This step was repeated a total of two times, the eluates were combined, adjusted to 0.25 M NaCl and samples were incubated at 65°C overnight to reverse crosslinking. DNA was isolated from each sample using the Qiagen QIAquick PCR purification kit (Valencia, CA) according to the manufacturer's specifications. Purified DNA samples were stored at −20°C. Quantitative PCR analysis was performed with equivalent amounts of purified DNA according to manufacturer's specification (LightCycler 480 DNA SYBR Green kit and LightCycler 480 system; Roche Diagnostics, Indianapolis, IN). GAPDH was used as the housekeeping gene. qRT-PCR assays were performed in triplicate and were repeated three times. The primers used to characterize the *Tnf* gene promoter were located in the: TATA box proximal region: 5′-CAC ACA CAC CCT CCT GAT TG-3′ and 5′-AGA CGG CCG CCT TTA TAG C -3′ generating fragments spanning nucleotides (nts) −19 to −276; and in the distal region 5′-GAG GCT CCG TGG AAA ACT CA-3′ and 5′ GCA GAG CAG CTT GAG AGT TGG GAA-3′ generating fragments spanning nts −546 to −756. The primers used to characterize the *Tnf* gene coding region were: 5′-TAT GGC TCA GGG TCC AAC TC-3′ and 5′-TGG TCA CCA AAT CAG CGT TA-3′ generating fragments spanning nts +1441 to +1537. All ChIP assays were repeated a minimum of three times.

### Real-time PCR

Control and treated cell monolayers were rinsed with ice cold PBS and collected by centrifugation (1,310× g, 4 min, 4°C). PBS was removed and total cellular RNA was isolated using the RNeasy Plus Mini Kit (Qiagen Inc., Valencia, CA). RNA (2 µg) was subject to reverse transcription with random primers using SuperScript III reverse transcriptase (Invitrogen). qRT-PCR was performed with equivalent amounts of cDNA according to manufacturer's specification (LightCycler 480 DNA SYBR Green kit and LightCycler 480 system; Roche Diagnostics, Indianapolis, IN). GAPDH was used as the housekeeping gene. qRT-PCR assays were performed in triplicate and were repeated three times. The following primers were used to amplify different regions of the *Tnf* gene (initiation, +24–+121): 5′-TAG CCA GGA GGG AGA ACA GA-3′ and 5′-TTT TCT GGA GGG AGA TGT GG-3′; (elongation, +1441–+1537): 5′-CAC CTG GCC TCT CTA CCT TG-3′ and 5′-GAC AGC CTG GTC ACC AAA TC-3′.

### Microarray analysis

Total cellular RNA, isolated from untreated and TNFα treated (rhTNFα 10 ng/ml, 1 h) SIMPL^−/−^ or littermate control MEFs was converted to cRNA and used to identify expressed genes (IU Center for Medical Genomics Microarray Core, Affymetrix Mouse Gene 1.0 ST GeneChip®). Data was extracted with Affymetrix Microarray Suite version 5 software and exported to a MicroArray Data Portal in the Core Facility. MEFs were derived from a single mouse embryo a total of 4 independent times for each strain (littermate, SIMPL^−/−^) and each set of fibroblasts was analyzed individually enabling us to detect small differences (≥1.5-fold) in gene expression with good statistical significance; data were corrected for false discovery rate [26]. Raw and processed microarray data are available in the National Center for Biotechnology Information Gene Expression Omnibus repository (http://www.ncbi.nlm.nih.gov/geo, accession number GSE44146).

### Progenitor and competitive repopulation assays

Bone marrow was isolated from 8–12 week old littermate or SIMPL^−/−^ mice (C57BL/6 background), cells were pelleted and resuspended in Iscove's Modified Dulbecco's Media. Cells were plated at a density of 1×10^4^ cells/mL in a methylcellulose mix containing: FBS (30%), penicillin/streptomycin (2%), β-mercaptoethanol, glutamine (1%), mSCF (100 ng/mL), 5% (vol/vol) pokeweed mitogen mouse spleen cell conditioned media and for BFU-E and CFU-GEMM, hemin (0.1 mM) and erythropoietin (1 unit/ml). Plated cells were incubated in a 5% oxygen tension incubator and colonies were counted 7 days later. Individual assays were performed in triplicate with a total of 9 SIMPL^−/−^ mice and 9 littermate control mice analyzed.

To characterize the hematopoietic stem cell (HSC) pool, competitive repopulation assays were performed as described by [27]. Briefly, bone marrow cells isolated from SIMPL^−/−^ mice (C57BL/6 background) or littermate control mice (C57BL/6 background), positive for the surface marker CD45.2^+^ were mixed (1∶1) with congenic CD45.1^+^ competitor bone marrow cells obtained from B6.SJL-Ptprc^a^ Pepc^b^/BoyJ mice (B6.BoyJ) (Jackson Labs). Cell mixtures were transplanted intravenously into lethally irradiated (1100-cGy split dose) B6.BoyJ mice. A total of ten B6.BoyJ mice were transplanted: 5 recipient B6.BoyJ mice received cell mixtures containing bone marrow cells isolated from the SIMPL^−/−^ mice; 5 recipient B6.BoyJ mice received cell mixtures containing littermate control bone marrow cells. Contribution to peripheral blood chimerism was assessed by measuring the proportion of CD45.2^+^ and CD45.1^+^ cells at multiple time points for 12 months post-transplant.

### Ethics Statement

This study was carried out in strict accordance with the recommendations in the Guide for the Care and Use of Laboratory Animals of the National Institutes of Health. All studies were approved by the Institutional Animal Care and Use Committee of Indiana University School of Medicine (Protocol 10295).

### Statistical analysis

Unless indicated otherwise all data were analyzed using an unpaired, 2-tail t-test and are presented as the average +/− SEM.

## Results

### SIMPL is required for a subset of TNFα-induced NF-κB dependent genes

**Figure 1:**
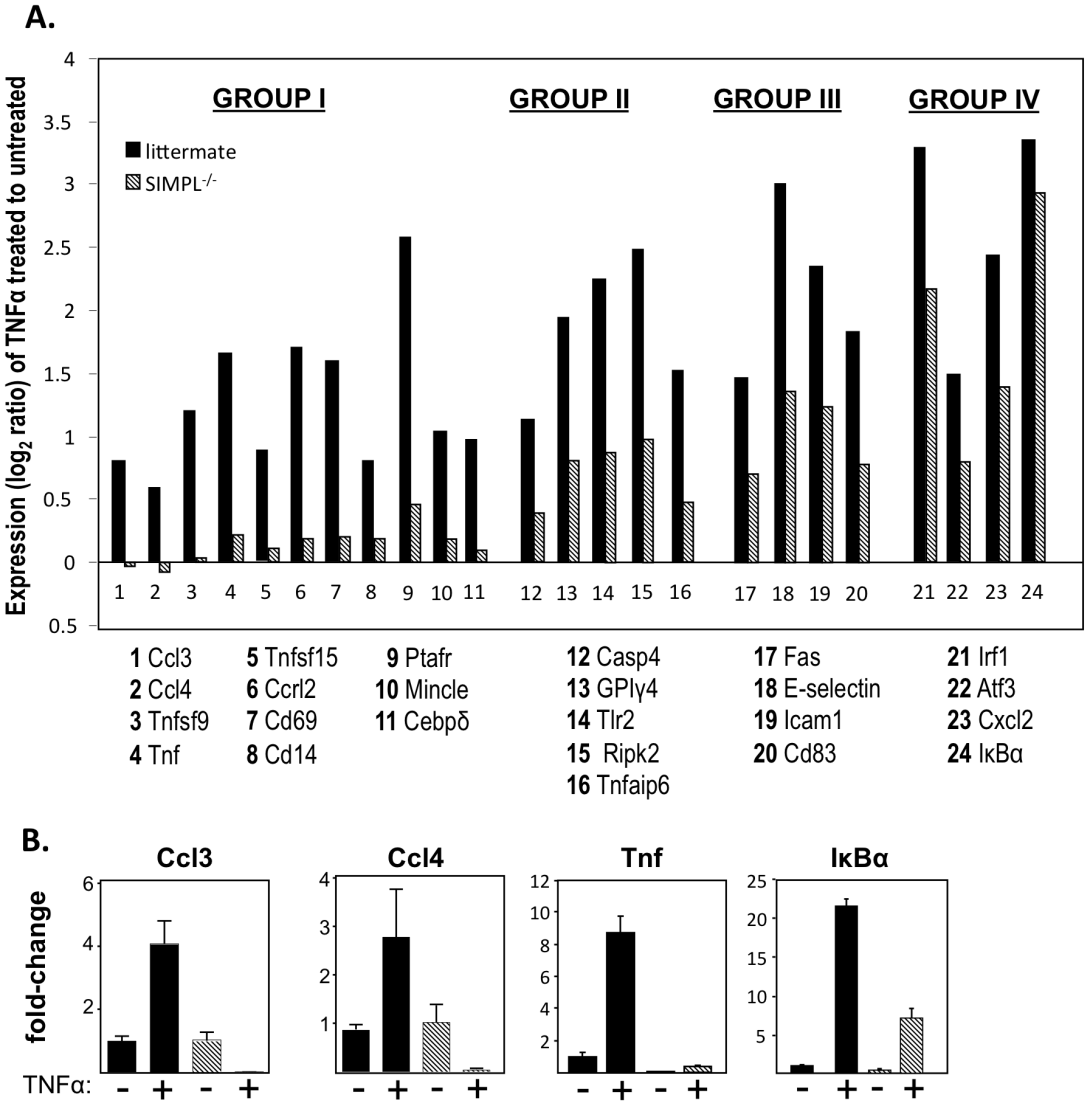
Identification of SIMPL-dependent genes. (**A**) Expression level of genes in TNFα treated as compared untreated MEFs derived from either littermate (▪) or SIMPL^−/−^ (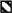) mice. Genes are grouped according differences in TNFα responsiveness of treated SIMPL^−/−^ derived as compared to littermate derived MEFs: complete loss (Group I); 75% reduction (Group II); 50% reduction (Group III) or a 25% reduction (Group IV). (**B**) MEFs derived from littermate (▪) or SIMPL^−/−^ (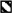) mice were treated with rhuTNFα (10 ng/ml) for 45 minutes, total RNA was isolated, converted to cDNA for analysis of indicated mRNAs by qRT-PCR.

Despite significant advances in the identification of the upstream activators of NF-κB, the regulatory mechanisms governing signal-specific changes in NF-κB dependent gene expression are comparatively less well understood [3],[16]. SIMPL is a key regulator of TNFα, but not IL-1 induced NF-κB activity [20], [21], identifying SIMPL as a unique target to probe for signal specific NF-κB regulation. Therefore, we performed a microarray analysis on RNA isolated from TNFα treated MEFs derived from littermate control or SIMPL^−/−^ mice (**Fig. 1**). Rank order differences identified 174 genes significantly induced >1.5-fold (p<10^−3^) in the TNFα treated littermate control MEFs. Of the 174 genes, 34 genes were either not induced (**Fig. 1A**, Group 1 genes) or were expressed at significantly reduced levels in the TNFα treated SIMPL^−/−^ MEFs (**Fig. 1A**, representative genes in Groups 2–4). Changes in several of the TNFα-induced SIMPL dependent genes were confirmed by qRT-PCR (**Fig. 1B**). The subset of 11 TNFα-induced SIMPL-dependent genes are known NF-κB dependent genes [28]–[32] whose products play a role in the recruitment and activation of hematopoietic cells (TNFα itself; Tnfsf9; Ccl3, Ccl4); pathogen recognition (Ptafr, Mincle); leukocyte adhesion (Ccrl2, Cd69, Cd14); a regulator of endothelial proliferation (Tnfsf15) and a transcriptional regulator (Cebpδ). The biological activities associated with the subset of SIMPL dependent-TNFα-induced genes links SIMPL to the inflammatory response.

### SIMPL is required for steady-state hematopoietic progenitor and stem cell function

**Figure 2:**
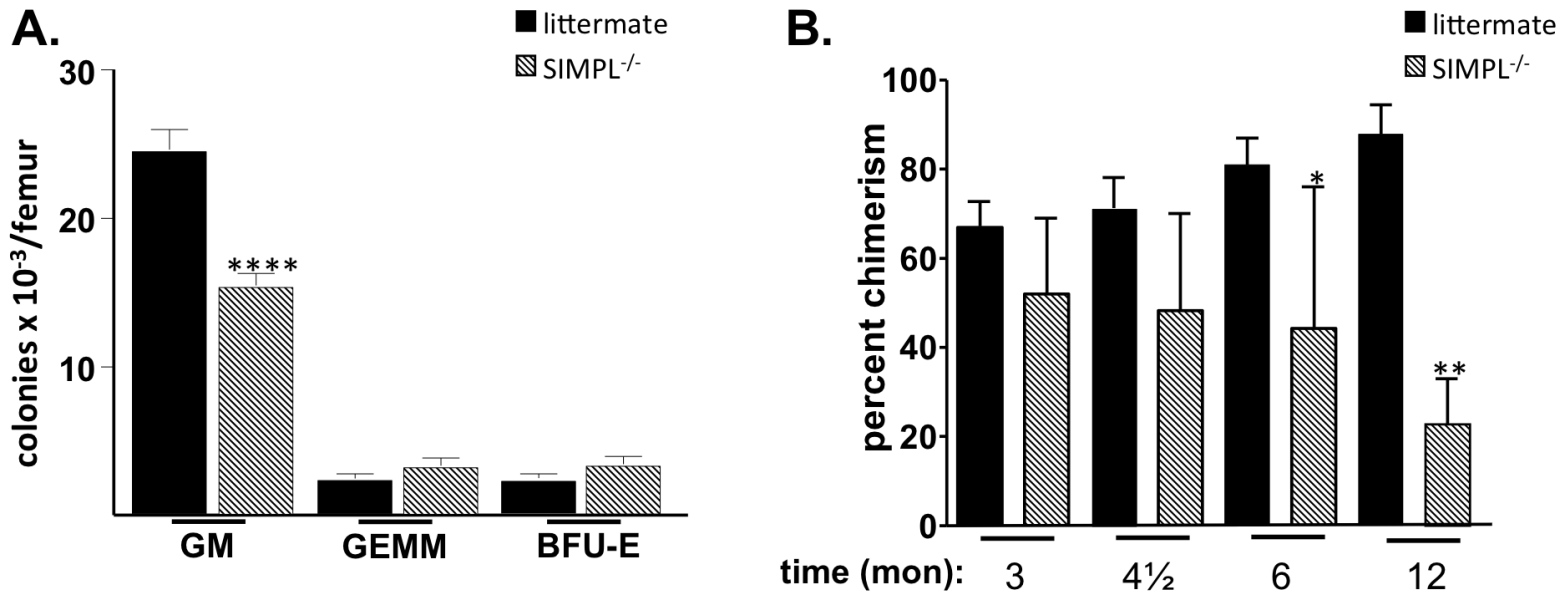
SIMPL is required for steady-state hematopoietic progenitor and stem cell function. (**A**) Low density bone marrow cells were cultured in triplicate in clonogenic methylcellulose progenitor assays at 1×10^4^ cells/mL. The total number of colony forming units (CFU; CFU-GM; BFU-E or CFU-GEMM) were quantitated 7 days later. A 2-tailed t-test was used to determine statistical significance with individual assays performed in triplicate (n = 9 animals/per strain) (**B**) Whole bone marrow cells from SIMPL^−/−^ or littermate controls (CD45.2^+^) were mixed with congenic CD45.1^+^ competitor marrow cells (1∶1) in a 1∶1 ratio (200,000 cells from each) and transplanted intravenously into lethally irradiated CD45.1^+^ mice (1100-cGy split dose). The proportion of CD45.1^+^ and CD45.2^+^ cells in peripheral blood was determined monthly by flow cytometry. Percent chimerism reflects the proportion of CD45.2^+^ cells present in the peripheral blood. A 2-tailed t-test was used to determine statistical significance (n = 5 animals/per strain, each assayed individually), ****p<0.0001; **p<0.01; *p<0.05.

During an inflammatory response, hematopoietic progenitors are recruited into the cell cycle and stimulated to undergo differentiation. Given the importance of TNFα as a regulator of steady-state hematopoiesis [33]–[35], we next examined whether hematopoietic cell function was altered in the SIMPL^−/−^ mice. Bone marrow cells from SIMPL^−/−^ and littermate control mice were plated in semi-solid culture in the presence of growth factors appropriate for enumerating the presence of 3 different types of hematopoietic progenitor cells (HPC). In these assays, a colony is derived from a single progenitor cell that, depending upon the cytokines present, undergoes proliferation and differentiates into a colony containing granulocytes and macrophages (CFU-GM), erythroid cells (BFU-E) or granulocytes, macrophages, erythroid cells and megakaryocytes (CFU-GEMM, a multipotential progenitor preceding CFU-GM and BFU-E). Analysis of cells from the SIMPL^−/−^ mice revealed a significant decrease in the total absolute number of CFU-GM per femur and no change in the numbers of BFU-E or CFU-GEMM per femur (**Fig. 2A**).

To characterize the hematopoietic stem cell (HSC) compartment, competitive repopulation assays were performed as described under Materials and Methods. Analysis of the transplanted mice revealed a significant decrease in the competitive repopulating ability of the HSCs lacking SIMPL at 6 and 12 months post-transplant (40% and 70% decrease, respectively; **Fig. 2B**). Thus, deletion of SIMPL results in compromised hematopoiesis, as indicated by losses in HPC and HSC function, a phenotype also seen in TNF RI^−/−^ mice [34].

### SIMPL localization to the TNFα gene promoter

**Figure 3:**
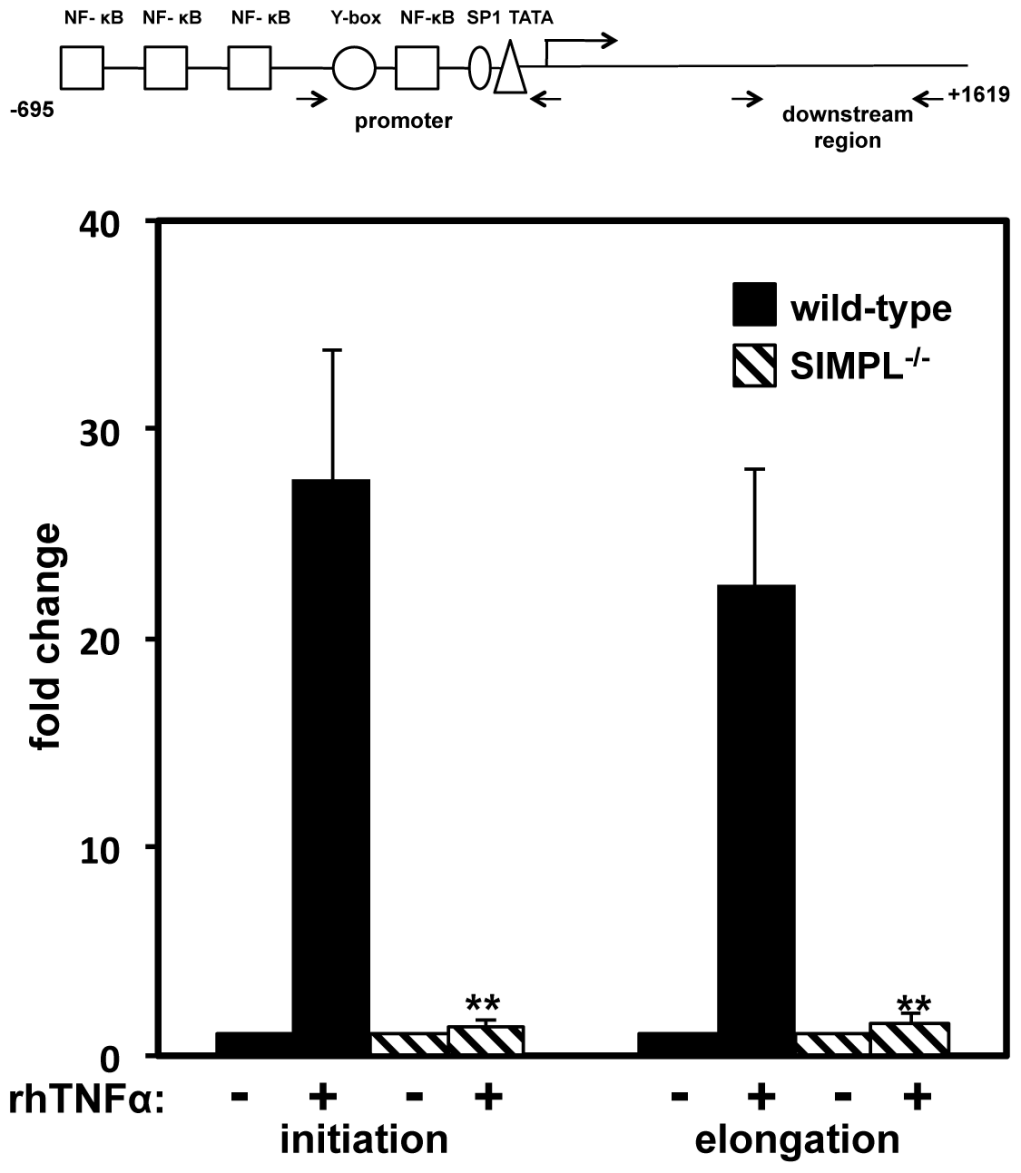
TNFα induces re-localization of SIMPL to the *Tnf* gene promoter. (**A**) Schematic of the mouse *Tnf* gene indicating location of PCR primers. (**B**) WT MEFs transfected with FLAG-SIMPL or empty FLAG-vector (Flag) and were left untreated (-) or were treated with rhTNFα (10 ng/ml; 45 min). Immunocomplexing assays performed with either Flag or p65 antibody were subjected to Western analysis with SIMPL antibody to confirm the TNFα-dependence of the p65-SIMPL interaction. LC-light chain. (**C**) WT MEFs were transfected with the indicated plasmids; 72 h post-transfection, chromatin immunoprecipitation (ChIP) assays were performed as described under Materials and Methods with the indicated antibodies and primer sets specific for the mouse *Tnf* gene (see Panel A for primer location; Materials and Methods for primer sequence). ChIP assays and corresponding quantitative real-time PCR assays were repeated at least three times.

TNFα signaling through the type I TNFα receptor stimulates the formation of nuclear SIMPL-p65 complexes where SIMPL synergistically enhances p65 trans-activation activity [20]. To investigate SIMPL's role in regulation of endogenous gene expression, Chromatin immunoprecipitation (ChIP) assays were used to examine whether SIMPL could be found at the TNFα gene promoter (see **Fig. 3A** for schematic). We focused on the *Tnf* gene as it is a known NF-κB regulated gene and our data (**Fig. 1**) along with the work of others [36] has shown that TNFα induces *Tnf* gene transcription. While we have generated SIMPL antibody [20] they have not worked in ChIP assays (data not shown). Thus, in these experiments wild-type MEFs were transfected with either empty expression vector or an expression vector encoding Flag-tagged wild-type SIMPL. Forty-eight hours later transfectants were either left untreated or were treated with rhTNFα (10 ng/mL) for 45 minutes. To confirm that TNFα regulated responses were being measured duplicate sets of transfected cultures were subjected to immunocomplexing assays. Results of these assays confirmed that Flag-SIMPL-p65 complex formation was not occurring in the absence of TNFα-treatment (**Fig. 3B**). ChIP assays were performed as described under Materials and Methods. Antibody that recognizes either p65 or the Flag-epitope was used to isolate DNA-containing complexes. The isolated DNA was used in quantitative real-time PCR reactions to quantify p65 and Flag-SIMPL binding to the mouse *Tnf* gene. In response to TNFα treatment, SIMPL and p65 were recruited to the region of the *Tnf* gene proximal to the TATA box whereas only p65 was recruited to the more distal region of the *Tnf* gene promoter (**Fig. 3C**). Neither SIMPL nor p65 were detected in the coding region of the *Tnf* gene. These assays reveal that in response to TNFα, SIMPL relocalizes to the *Tnf* gene promoter, where it preferentially associates with the region containing the TATA box proximal NF-κB binding sites.

### SIMPL enhances initiation of *Tnf* gene transcription

**Figure 4:**
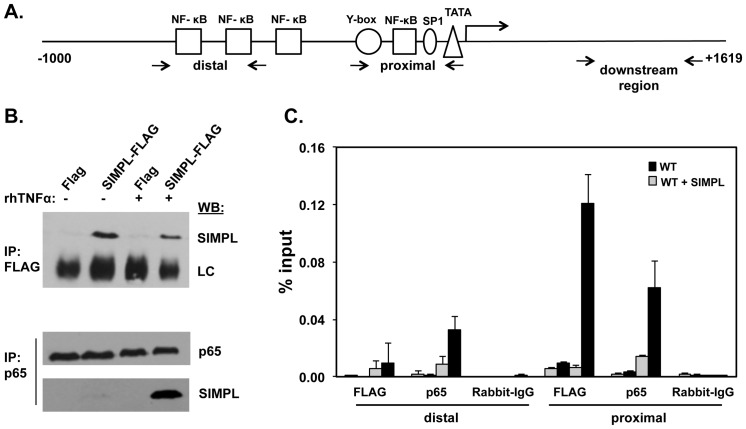
SIMPL is required for initiation of *Tnf* gene transcription. (**A**) Schematic of mouse *Tnf* gene annotated with the regions amplified by primers used to distinguish initiation (+24 to +121) and elongation (+1441 to +1537). (**B**) Total cellular RNA extracted from untreated and TNFα treated (rhTNFα 10 ng/ml, 45 min) MEFs derived from littermate control (WT) or SIMPL^−/−^ mice was subject to quantitative RT-PCR with the indicated primers. Fold-change represents a ratio of TNFα treated to untreated sample of the same genotype. Assays were repeated at least three times and a 2-tailed T-test was used to determine statistical significance, **p<0.01.

The localization of SIMPL to the TATA box proximal region of the *Tnf* gene promoter, combined with the microarray results revealing that SIMPL is required for the expression of a subset of TNFα induced genes, suggests that SIMPL modulates the initiation phase of transcription. To address this possibility we continued our focus on the *Tnf* gene (**Fig. 4A** **schematic**). TNFα treatment of wild-type MEFs leads to an increase in the number of *Tnf* gene transcripts detected with primers located either at the beginning (+24 to +121) or end (+1441 to +1537) of the *Tnf* gene transcript (**Fig. 4B**). In contrast and independent of the set of primers used, *Tnf* transcripts were not detected in either the control or TNFα treated MEFs lacking SIMPL (**Fig. 4B**). These data reveal that SIMPL is required for the initiation of TNFα induced *Tnf* gene transcription.

### SIMPL binding partners include components of the transcriptional apparatus

Signal dependent changes in gene transcription are mediated through a coordinated process involving a number of large, multi-subunit protein complexes. To gain insight into the role that SIMPL plays in regulation of p65 dependent gene expression mass spectrometry was used to identify SIMPL binding proteins isolated using the InterPlay™ Mammalian TAP system (Stratagene, La Jolla, CA) as described under Materials and Methods. A number of proteins known to be involved with the regulation of gene transcription were identified as novel SIMPL binding partners. Of particular interest was the identification of Mediator subunits as well as the second largest subunit of RNA polymerase II (Rpb2). Localization of SIMPL in complexes containing these proteins could explain the profound enhancement of p65 trans-activation activity induced by SIMPL using the Gal4 system [20].

### SIMPL-p65-MED1 complex formation

**Figure 5:**
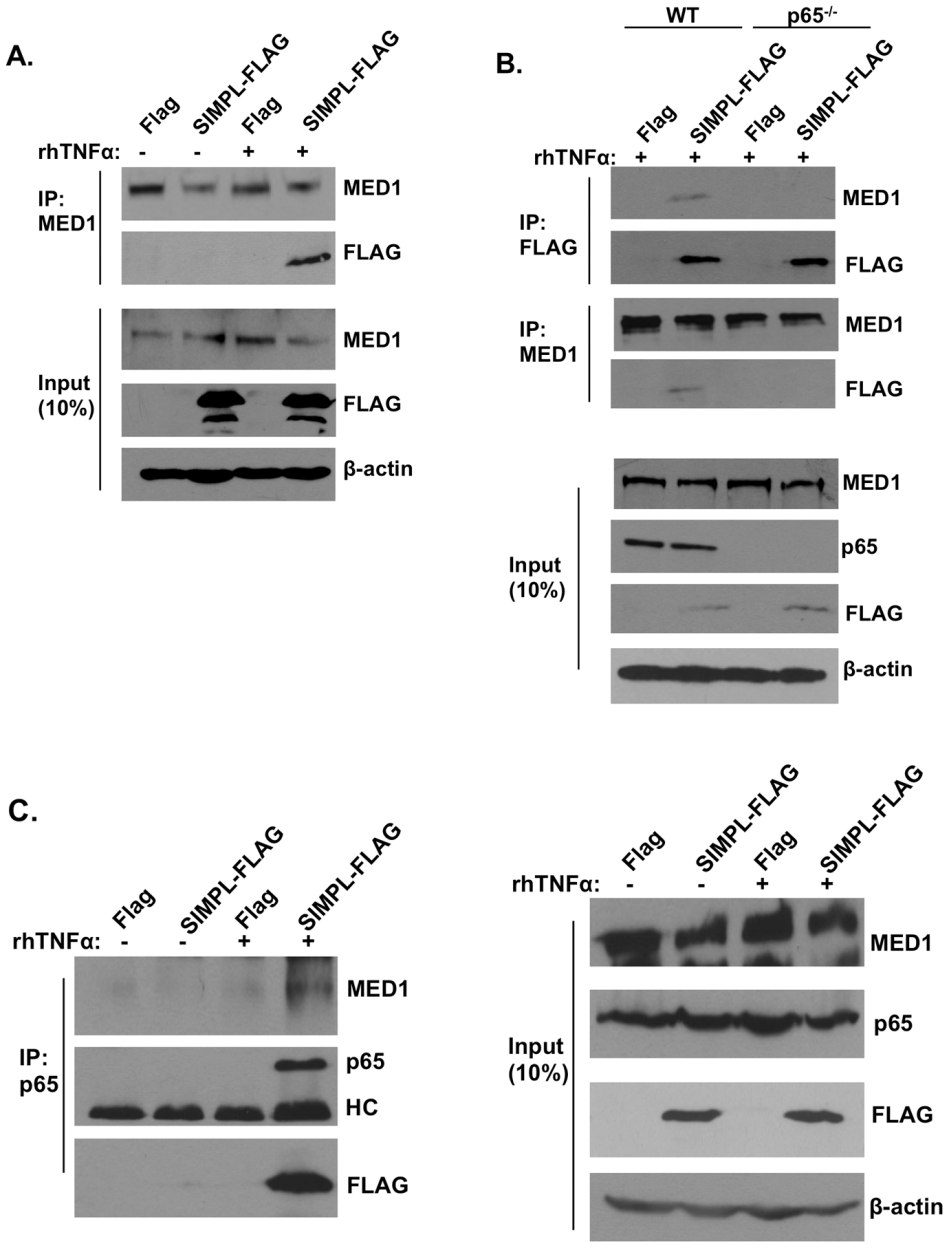
TNFα induced formation of SIMPL-MED1 complexes is p65 dependent. (**A**) HEK 293 cells or (**B**) wild-type MEFs or MEFs lacking p65 were transfected with SIMPL-FLAG or empty FLAG-vector, 72 h later cells were left untreated (-) or treated with rhTNFα (10 ng/ml) for 45 min. Upper panels: immunocomplexing assays were performed with antibody to MED1 (IP:MED1) and/or FLAG (IP:FLAG) and immunocomplexed materials were subjected to Western analysis with the antibody to the proteins indicated on the right hand side of the figure. Lower panels: Cell lysates (50 ug) used in immunocomplexing assays were subjected to Western analysis with antibodies to the indicated proteins. (**C**) HEK 293 cells were transfected with the indicated constructs, 72 h later cells were left untreated or treated with rhTNFα (10 ng/ml) for 45 min. Cell lysates were either analyzed directly (panel labeled input) or were subjected to immunocomplexing assays with Flag-antibody coupled sepharose beads, materials bound to the agaroase beads were eluted with Flag peptide and subjected to immunocomplexing assays with antibody to p65. Immunocomplexed materials were subjected to Western analysis with the antibodies to the proteins indicated on the right hand side of the figure. All assays were repeated at least three times.

Since MED1 had been linked to hematopoietic cell function [37], [38] we examined in more detail the interaction between SIMPL and MED1. HEK 293 cells were transfected with an empty expression vector or a vector encoding Flag-tagged wild-type SIMPL and 72 h later cell monolayers were stimulated with rhTNFα (10 ng/mL) for 45 minutes. After generating cell lysates, antibody recognizing MED1 was used to generate immunocomplexes that were subjected to Western analysis with antibody recognizing either the Flag-epitope or MED1. In the absence of TNFα, Flag-SIMPL was not detected in immunocomplexes generated with the MED1 antibody (**Fig. 5A**). However, in response to stimulation with TNFα, Flag-SIMPL was detected in the MED1 containing immunocomplexes (**Fig. 5A**). To determine whether p65 was required for the TNFα-induced SIMPL-MED1 complex formation, immunocomplexing assays, using MED1 (or Flag) antibody, were repeated in MEFs lacking p65 (**Fig. 5B**). Independent of the antibody used to generate the immunocomplexes (MED1 or Flag), in the absence of p65, TNFα treatment did not lead to the generation of immunocomplexes containing SIMPL and MED1. Results of these assays revealed that p65 is required for TNFα-induced SIMPL-MED1 complex formation.

Results of the immunocomplexing assays strongly suggested that TNFα stimulated the formation of complexes containing SIMPL, p65 and MED1. To directly test this hypothesis HEK 293 cells were transfected with an empty expression vector or a vector encoding Flag-tagged SIMPL. 72 h later cell monolayers were stimulated with rhTNFα (10 ng/mL) for 45 minutes, cell lysates were generated and antibody recognizing the Flag-epitope conjugated to agarose beads was used to generate immunocomplexes. The immunocomplexed materials were subjected to three cycles of resuspension-centrifugation in tris-buffered saline (TBS; 50 mM Tris H-Cl, 150 mM NaCl). The final pellet was resuspended in TBS and flag-peptide was added to elute Flag-SIMPL and associated proteins. Antibody to p65 was added to the Flag-peptide-eluted materials, and the resulting immunocomplexes were subjected to Western analysis using MED1, p65 and Flag antibody (**Fig. 5C**). When the Flag-peptide eluted materials were subjected to immunocomplexing assays with p65 antibody, Flag-SIMPL and MED1 were detected only if the cell cultures were treated with TNFα. Thus in response to TNFα, complexes containing at least SIMPL, p65 and MED1 form.

### SIMPL enhances p65-MED1 complex formation

**Figure 6:**
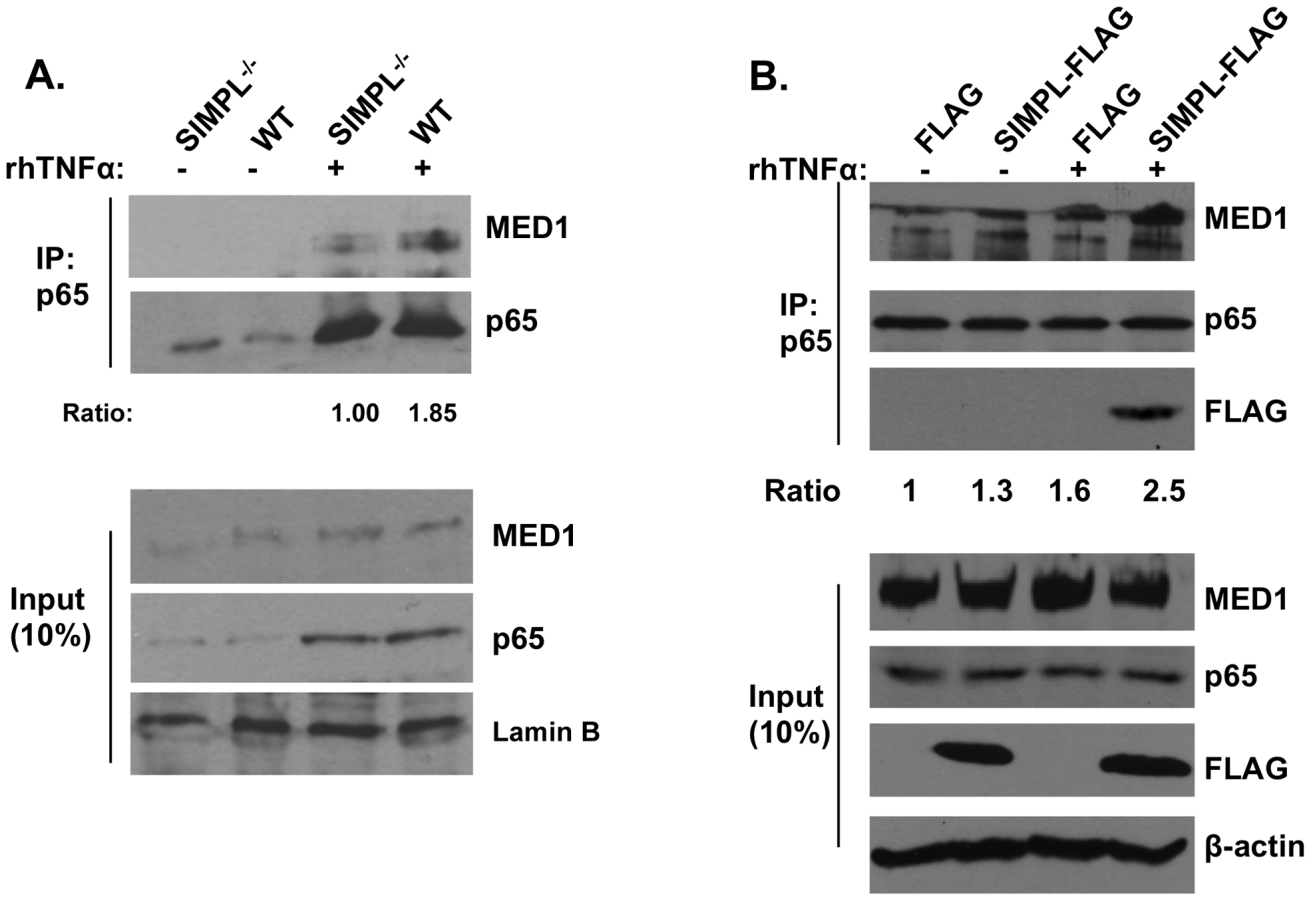
SIMPL enhances p65-MED1 complex formation. (**A**) MEFs derived from littermate controls (WT) or SIMPL^−/−^ mice were left untreated (-) or were treated with rhTNFα (10 ng/ml) for 45 minutes. Upper panel: nuclear extracts were subjected to immunocomplexing assays with p65 antibody and immunocomplexed materials were subjected to Western analysis with antibodies to the indicated proteins. Lower panel: Western analysis of cell lysates (10%; 50 ug) used in immunocomplexing assays with antibodies to the indicated proteins. (**B**) HEK 293 cells transfected with FLAG-SIMPL or empty FLAG-vector (Flag) and were left untreated (-) or were treated with rhTNFα (10 ng/ml; 45 min). Upper panel: immunocomplexing assays performed with p65 antibody were subjected to Western analysis with antibodies to the indicated proteins. Lower panel: Western analysis of cell lysates (10%; 50 ug) used in immunocomplexing assays with antibodies to the indicated proteins. n = 3.

The ability of SIMPL to induce the activity of a reporter plasmid under the control of trimerized NF-κB sites is dependent upon p65; whereas in the same assay the absence of SIMPL does not affect the activity of ectopically expressed p65 [20]. In the absence of SIMPL, TNFα-induced activation of a reporter under the control of trimerized NF-κB sites is reduced (∼80%) but not eliminated [20]. Taken together these data prompted us to examine whether SIMPL is required for p65-MED1 complex formation. MEFs derived from SIMPL^−/−^ mice, or the corresponding littermate controls were left untreated or were stimulated with rhTNFα (10 ng/mL) for 45 minutes. Cell lysates were generated and immunocomplexes generated with antibody recognizing p65 were subjected to Western analysis with MED1. In wild-type MEFs, under steady-state conditions MED1 was detected in the p65 generated immunocomplexes and in response to TNFα there was ∼2-fold increase in p65-MED1 complex formation (**Fig. 6A**). In cells lacking SIMPL, p65-MED1 complexes were detected under steady-state conditions and relative to the wild-type cells, a more modest increase in p65-MED1 complex formation detected in response to TNFα (∼1.4-fold). The effect of ectopically expressed SIMPL on p65-MED1 complex formation was also measured. HEK 293 cells were transfected with an empty expression vector or a vector encoding Flag-tagged wild-type SIMPL. 72 h later cell monolayers were stimulated with rhTNFα (10 ng/mL) for 45 minutes, cell lysates were generated and antibody recognizing p65 was used to generate immunocomplexes and the resulting immunocomplexes were subjected to Western analysis using MED1 and Flag antibody (**Fig. 6B**). Results of these assays revealed that increasing the amount of SIMPL protein enhanced the amount of TNFα-induced p65-MED1 complex formation (∼2-fold). Taken together, the data presented in Figure 6 reveal that SIMPL protein is not required for but instead enhances p65-MED1 complex formation.

**Figure 7:**
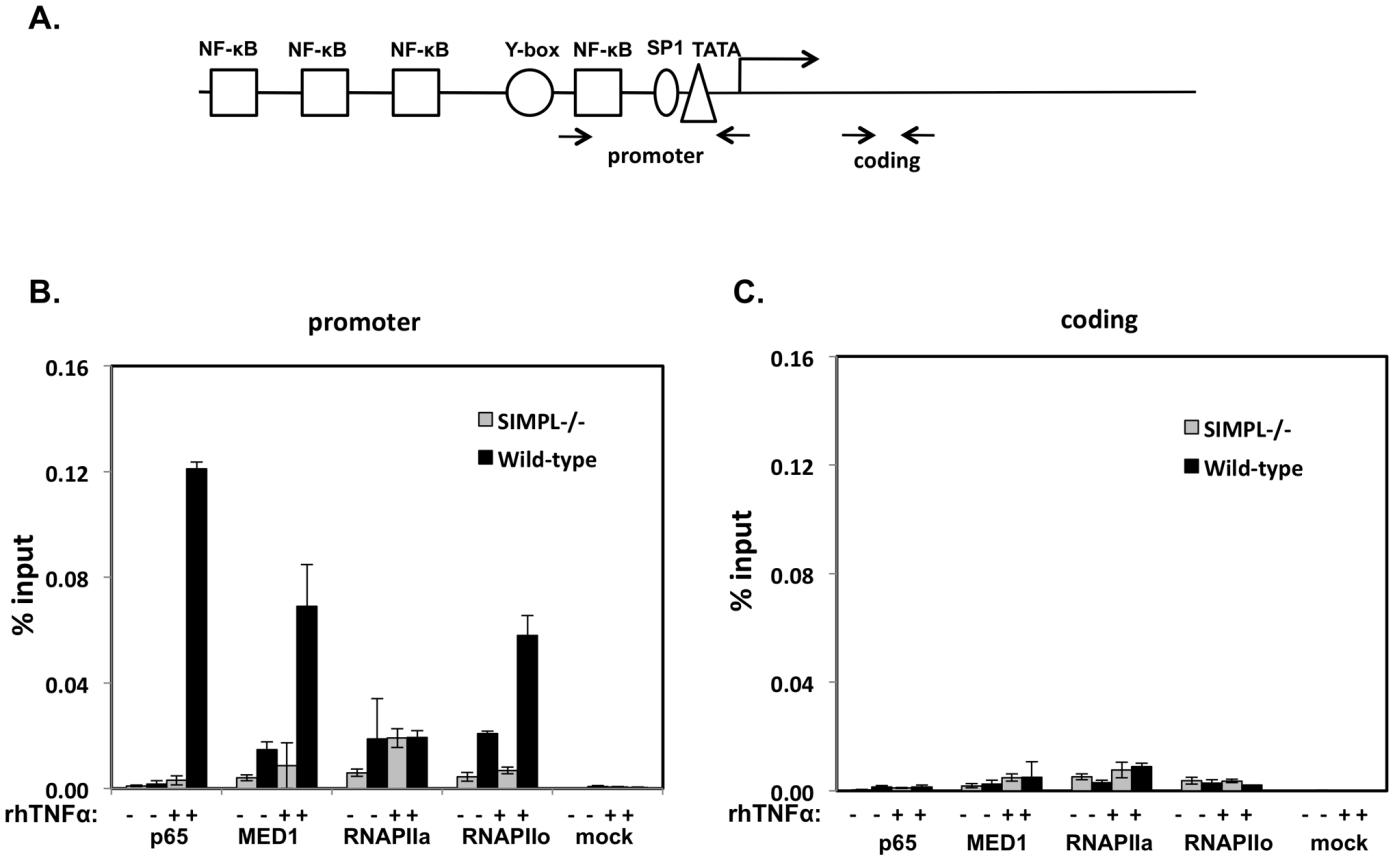
SIMPL enhances the appearance of p65, MED1 and serine 5 phosphorylated RNA pol II on the *Tnf* gene promoter. (**A**) Schematic of the mouse *Tnf* gene indicating location of PCR primers. (**B and C**) MEFs derived from littermate controls (WT) or SIMPL^−/−^ mice were left untreated (-) or were treated with rhTNFα (10 ng/ml) for 45 minutes. Chromatin immunoprecipitation (ChIP) assays were performed as described under Materials and Methods with the indicated antibodies and primer sets specific for the mouse *Tnf* gene (see Panel A for location of primers; Materials and Methods for sequence of primers). ChIP assays were repeated at least three times.

We next examined whether the appearance of either p65 or MED1 on the *Tnf* gene promoter required the presence of SIMPL. MEFs derived from wild-type or SIMPL^−/−^ mice were treated with TNFα for 45 minutes and processed for ChIP assays as described above (**see legend** **Fig. 3**). In response to TNFα there is increased association of p65, MED1 and phospho-RNA polymerase II (serine 5; RNAPIIo) associated with the mouse *Tnf* gene promoter in MEFs derived from wild-type mice (**Fig. 7B**); these changes were dramatically reduced in the MEFs derived from mice lacking SIMPL (**Fig. 7B**). Parallel ChIP assays were performed with sequences located in the TNFα coding region to confirm the specificity of the proteins detected at the *Tnf* gene promoter region (**Fig. 7C**). Together this series of experiments (**Figs. 5** **–** **7**) reveals that SIMPL serves as a transcriptional co-activator by enhancing in a TNFα-dependent manner, p65-MED1 complex formation.

## Discussion

Regulation of gene transcription in a cell-state and signal-specific manner requires the coordinated actions of numerous transcription factors, co-activators and regulators. Previous studies from our laboratory identified the novel regulatory factor SIMPL, and demonstrated that SIMPL interacts with p65 in a TNFα dependent manner where it specifically enhances p65 transactivation activity [20], [21]. We now have defined a unique regulatory mechanism for SIMPL involving localization to the *Tnf* gene promoter, in a signal specific manner, where it enhances p65 and MED1 complex formation. To our knowledge these are the first studies to demonstrate an interaction between endogenous p65 and endogenous MED1.

Mediator functions at several steps during transcription: RNA polymerase II recruitment, pre-initiation complex formation, post-recruitment processes as well as DNA loop formation (for review see [39]–[41]). The structural interactions and functional relationship between Mediator and steroid hormone receptors, and some mammalian transcription factors have been characterized. The interaction between Mediator and the c-Rel family of transcription factors is relatively less well understood. Analysis of HIV transcription demonstrated that a crude complex of Mediator proteins (containing MED1) significantly promoted p65 dependent transcription of the NF-κB dependent HIV-1 promoter [42]. Additional studies demonstrated that the p65 transactivation domain allowed for an association between Mediator and the HIV-LTR [43]. Work by others analyzing proteins bound to a GST-p65 transactivation domain fusion protein identified a number of mediator subunits including MED1 and MED15 [44]. MED1 and MED15 were also identified in SIMPL containing complexes in our analysis. Our immunocomplexing assays revealed that SIMPL is not required for p65-MED1 complex formation, but instead increases the abundance of endogenous p65-MED1 complexes.

Consistent with SIMPL functioning as a component of a TNFα signaling pathway, SIMPL^−/−^ mice, similar to mice lacking TNF RI, are viable. Previous studies using SIMPL shRNA suggesting that SIMPL may be required for HSC function [45] led us to examine the early immature hematopoietic cell compartments in the SIMPL^−/−^ mice. Immunophenotypic analysis of peripheral blood from the SIMPL^−/−^ mice and littermate controls did not reveal significant differences in the numbers of circulating hematopoietic cells (data not presented). However, assessment of bone marrow-derived progenitor cell function *in vitro* revealed a decrease number of granulocyte/macrophage progenitors in the SIMPL^−/−^ mice. Since the overall bone marrow cellularity does not differ between the SIMPL^−/−^ and littermate controls (data not shown), the decrease in progenitors reflects diminished numbers of progenitors and/or their proliferation. Resolving this difference will require additional study. Analysis of hematopoietic stem cell function confirmed that SIMPL is required for intrinsic hematopoietic stem cell function. These data parallel competitive repopulation assays performed with cells derived from mice lacking TNF RI [34]. Thus, SIMPL is a component of the TNF RI network intrinsic to hematopoietic stem cells and is necessary for proper hematopoietic maintenance.

TNFα also plays a central role in regulating host defense through its effects upon non-hematopoietic cells. In response to tissue damage and/or pathogens, a complex physiological response is initiated through activation of the innate immune response that in some instances will transition to activation of the adaptive immune response. The profile of SIMPL dependent/modulated TNFα-induced genes was determined following a one hour TNFα treatment. Loss of SIMPL affects TNFα dependent expression of genes whose products mediate pathogen recognition, immune cell recruitment, activation and proliferation, as well as transcription of additional inflammatory mediators. The biology associated with the subset of TNFα-induced genes that are SIMPL dependent is unclear. It may reflect host responsiveness to a specific pathogen(s) or a mechanism through which host TNFα levels are controlled.

In the last few decades modulation of the inflammatory response has been a major target for pharmaceutical development. While many of the newer pharmaceuticals targeting aspects of inflammation have been very effective, many are also associated with a significant risk of adverse events. To develop more selective agents, it is critical that insight into mechanisms that regulate signal specific and/or gene specific regulation of transcription is gained. Molecules like SIMPL provide one mechanism through which the activity of transcription factors, activated by numerous stimuli, can elicit distinct patterns of gene expression. Our studies are one of the first to identify a protein that can explain how changes in NF-κB dependent gene expression are controlled in a TNFα specific manner (data herein; [20], [21]). Moreover our studies describe a novel paradigm, signal-specific control of p65 transcriptional activity, for the development of therapeutic modalities that attenuate versus eliminate TNFα activity.

The link between SIMPL and hematopoiesis is intriguing. Myelodysplastic syndrome (MDS) is a group of complex disorders characterized by ineffective hematopoiesis (HSCs with compromised self-renewal ability) usually diagnosed later in life (>60 years). Typically early stage MDS patients have normal or elevated levels of TNFα, a negative regulator of hematopoiesis [33], [46]. Current models suggest as MDS evolves, resistance to the growth inhibitory effects of TNFα are lost, increasing the probability that a leukemic clone will develop [47]. The observation that the SIMPL^−/−^ mice share a phenotype in common with TNF RI^−/−^ mice strengthens our hypothesis that SIMPL is an integral member of the TNFα signaling pathway. Moreover, elucidating the molecular details of SIMPL function may identify therapeutic target(s) for the treatment of MDS.
